# Growing three-dimensional biomorphic graphene powders using naturally abundant diatomite templates towards high solution processability

**DOI:** 10.1038/ncomms13440

**Published:** 2016-11-07

**Authors:** Ke Chen, Cong Li, Liurong Shi, Teng Gao, Xiuju Song, Alicja Bachmatiuk, Zhiyu Zou, Bing Deng, Qingqing Ji, Donglin Ma, Hailin Peng, Zuliang Du, Mark Hermann Rümmeli, Yanfeng Zhang, Zhongfan Liu

**Affiliations:** 1 Center for Nanochemistry (CNC), Beijing Science and Engineering Research Center for Nanocarbons, Beijing National Laboratory for Molecular Sciences, State Key Laboratory for Structural Chemistry of Unstable and Stable Species, College of Chemistry and Molecular Engineering, Peking University, Beijing 100871, China; 2 Key Laboratory for Special Functional Materials (Ministry of Education), Henan University, Kaifeng 475004, China; 3 Department of Materials Science and Engineering, College of Engineering, Peking University, Beijing 100871, China; 4 Centre of Polymer and Carbon Materials, Polish Academy of Sciences, M. Curie-Sklodowskiej 34, Zabrze 41-819, Poland; 5 IFW Dresden, 20 Helmholtz Strasse, Dresden 01069, Germany; 6 School of Energy, Soochow University, Suzhou 215006, China

## Abstract

Mass production of high-quality graphene with low cost is the footstone for its widespread practical applications. We present herein a self-limited growth approach for producing graphene powders by a small-methane-flow chemical vapour deposition process on naturally abundant and industrially widely used diatomite (biosilica) substrates. Distinct from the chemically exfoliated graphene, thus-produced biomorphic graphene is highly crystallized with atomic layer-thickness controllability, structural designability and less noncarbon impurities. In particular, the individual graphene microarchitectures preserve a three-dimensional naturally curved surface morphology of original diatom frustules, effectively overcoming the interlayer stacking and hence giving excellent dispersion performance in fabricating solution-processible electrodes. The graphene films derived from as-made graphene powders, compatible with either rod-coating, or inkjet and roll-to-roll printing techniques, exhibit much higher electrical conductivity (∼110,700 S m^−1^ at 80% transmittance) than previously reported solution-based counterparts. This work thus puts forward a practical route for low-cost mass production of various powdery two-dimensional materials.

## Abstract (web summary)

High-volume, low-cost production of graphene is pivotal for the industrial advance of this 2D material. Here, the authors make use of naturally occurring diatomite as a 3D substrate for graphene growth, obtaining non-planar porous graphene structures after removal of the silica templates.

## Graphical abstract

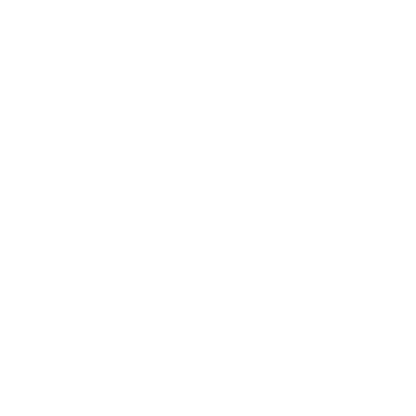

One of the keys for determining the industrial future of graphene material is its mass production technology with high quality and low cost^1^,^2^,^3^,^4^. Currently, there are two typical routes for the scalable production of graphene, giving graphene powders or films^5^,^6^,^7^,^8^,^9^,^10^. The graphene powders are usually synthesized from graphite powders based on wet chemistry approaches, including reduced graphene oxide (RGO) and liquid-phase exfoliated graphene (LPEG)^11^,^12^. The production ability of such chemically exfoliated graphene powders has reached up to a level of kilotons per year with relatively low cost. However, the chemically exfoliated graphene materials are generally rich of structural defects and chemical residues such as –OH, –C=O, –COOH and other noncarbon impurities, which undesirably lose the fascinating properties of pristine graphene^13^,^14^,^15^. The homogeneous high-concentration dispersion of such graphene powders in solution is still of great technical challenge due to the strong π–π stacking interactions^16^ between planar graphene sheets. This severely limits their solution processability and retards various applications of graphene powders.

Another route to mass production relies on chemical vapour deposition (CVD) techniques, which generate high-quality graphene films^8^,^9^,^17^,^18^,^19^. The current CVD graphene films grown on catalytic metal surfaces have become competitive in crystallinity with mechanically exfoliated graphene, together with sub-million square metres per year yield. Such kinds of high-quality graphene films would find wide applications in electronic and optoelectronic devices^20^,^21^. However, these graphene films need to be transferred from the metal foils onto target substrates through the conventional wet chemical etching method, which inevitably results in contaminant adsorption on graphene, degradation of graphene intrinsic properties, as well as consumption of metal foils.

An interesting question is whether we can combine the advantages of CVD graphene and chemically exfoliated graphene together, and develop a practical mass production route to high-quality graphene powders with low cost. For this purpose, we paid our attention to the naturally abundant biotemplates for CVD growth of graphene powders. The possible candidate along this consideration is industrially widely used diatomite^22^,^23^. Diatomite is a naturally existing material with great abundance on earth (about two million tons per year all over the world), originating from the ancient diatoms as the deposit of diatom frustules^24^. The chemical composition of diatomite is dominantly SiO_2_ (80–94%) with chemically removable trace amounts of metal oxides and organics. Diatomite has extremely high porosity with light weight, arising from the hierarchical porous structures of individual diatom frustules, which has been widely used as adsorbent, thermal insulating material, filter, catalyst support, decolouring agent, filling material and so on^24^,^25^,^26^.

In this work, we demonstrate the outstanding performance of diatomite as the template for growing hierarchically structured graphene powders by a surface-oxygen-assisted, small-methane-flow CVD route following a self-limited growth mechanism. Graphene growth on planar SiO_2_ substrates has been well known with good crystalline quality and much less noncarbon elements as compared with the chemically exfoliated graphene powders^27^,^28^. Hence, the presented approach based on naturally abundant diatomite is believed to be feasible in principle, giving a route for synthesizing graphene powders with high crystallinity and low cost. Notably, the synthesized graphene products are expected to be significantly different from the porous-silica-templated CVD graphitic carbon powders reported in the past decade^29^,^30^,^31^,^32^. In those CVD processes, the non-selective deposition and non-inductive nucleation of carbon species both on the surface and inside pores of silica templates (for example, zeolites and silica opals) usually lead to formation of the massive graphitic and amorphous coke particles. Morphologically inverse graphitic frameworks with respect to porous templates were retained after the removal of templates. In contrast, our CVD-grown graphene powders are expected to inherit the hierarchical structures of individual diatom frustules, creating a type of three-dimensional (3D) hierarchical biomorphic graphene (HBG). After the removal of silica templates, the non-planar porous structures of graphene flakes could be retained to be free from strong van der Waals and π–π stacking, and accessible to the solvent molecules, enabling a rapid and stable high-concentration dispersion of these graphene flakes in low boiling point, environment-friendly solvents (for example, ethanol). It may offer a practical solution to the serious aggregation problem encountered in chemically exfoliated graphene powders. Taking into account of the hundreds of versatile biological structures from different diatom sources, such kinds of hierarchical biomorphic graphene are designable in fine microstructures, depending on the purpose of practical applications. Besides, the graphene coating also provides a route for decorating the diatomite material with fascinating functions, which may further expand the wide industrial applications of diatomite itself.

## Results

### CVD growth of graphene powders on diatomites

**Figure 1:**
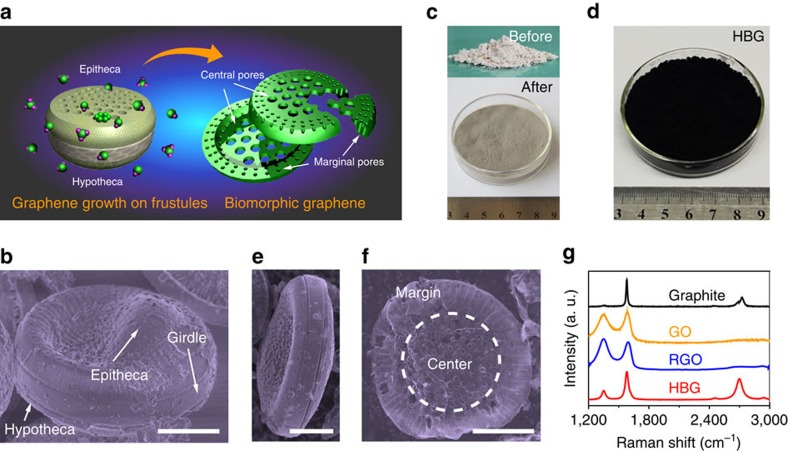
Diatomite-based CVD growth of graphene powders. (**a**) Schematic diagram showing graphene growth on a diatom frustule and its hierarchical biomorphic structures. (**b**) False colour SEM image of a diatom frustule. (**c**,**d**) Photographs of diatomite before and after graphene growth, as well as after removal of biosilica templates, respectively. (**e**,**f**) False colour SEM images of an isolated diatom frustule after graphene growth and an individual graphene flake, respectively. (**g**) Raman spectra of diatomite-derived graphene powders together with graphite, GO and RGO powders. Scale bars, 10 μm.

Diatomite has versatile microstructures depending on the diatom source, or say, producing area. Here we use *Coscinodiscus wailesii* diatom source for demonstration. An individual *Coscinodiscus wailesii* diatom frustule is composed of two almost equal valves (named as epitheca and hypotheca, respectively) fitting together with a girdle (a circular band of silica), and looks like a Petri dish^24^ (Fig. 1a,b). These valves, comprised of a porous layer of silica, can be separated with girdles by cycled settling from the frustule assemblies (Supplementary Fig. 1). The obtained porous biosilica powders were employed as the CVD growth templates of graphene (Supplementary Fig. 2). The fine hierarchical structures of such kinds of biosilica templates are expected to be preserved during the high-temperature CVD process, and further inherited by the derived graphene (Fig. 1a).

CVD growth of graphene on such biosilica templates changed the original white powders to light grey in macroscopic quantity (Fig. 1c). Removing the biosilica templates by wet-etching, we obtained the black graphene powders (Fig. 1d), accompanied with a marked weight loss. The morphologies of original diatom frustules were found to be well preserved after 1,000 °C growth process in the resulting individual graphene flakes (Fig. 1e,f; Supplementary Fig. 3). Raman spectrum (Fig. 1g) of thus-produced graphene powders exhibits two characteristic peaks of graphene at 1,582 cm^−1^(G band) and 2,700 cm^−1^(2D band), respectively. Remarkably different from the very weak and broad 2D peak of graphite, as well as the hardly observed 2D peak of RGO, the appearance of sharp 2D peak should indicate the formation of thin-layer graphene with less basal plane defects. The presence of weak Raman D band located at 1,350 cm^−1^ is attributed to the boundary defects of polycrystalline graphene powders with small domain sizes^33^,^34^. In brief, the above experimental observations confirmed the feasibility of diatomite-based approach for growing high-quality graphene powders. Powder X-ray diffraction patterns of these graphene powders also indicate the absence of layered (002) periodic structures of graphite (Supplementary Fig. 4), distinct from those of RGO and graphitic carbon powders^29^,^30^,^32^.

### Characterization of 3D biomorphic graphene

**Figure 2:**
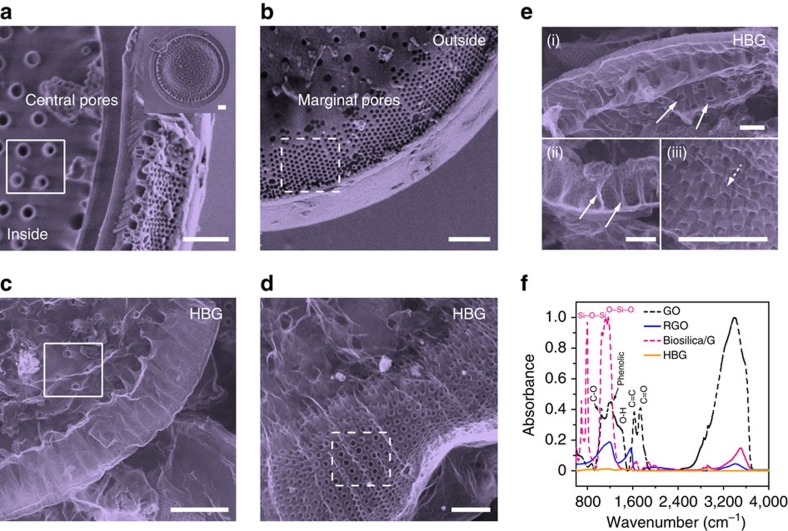
Hierarchical biomorphic structures of diatomite-derived graphene flakes. (**a**,**b**) False colour SEM images of the central and edge areas of a diatom frustule, respectively. The inset shows the whole valve image. (**c**,**d**) False colour SEM images of the derived HBG flakes corresponding to **a** and **b** after removal of diatom frustule. (**e**) Morphology of a HBG flake (i), cross-section of its central pores (ii) and details of its marginal pores (iii). (**f**) FTIR micro-spectra of HBG, Biosilica/G, RGO and GO powders. Scale bars, 2 μm.

Perfect replication of the fine structures from diatom frustules to graphene flakes is demonstrated in Fig. 2 by detailed structural comparisons. Half valve of an individual diatom frustule is actually a natural quasi-two-dimensional microplate (∼35 μm in diameter and 1–2 μm in thickness) made of porous silica skeleton (diatom cell wall). It possesses two typical hierarchical pores, that is, central pores radially distributed at the valve centre with a diameter of ∼800 nm and marginal pores quasi-periodically located at the valve edge with a diameter of ∼200 nm (Fig. 2a,b). Similar microscopic pore structures can be clearly seen in the graphene flakes after high temperature growth and removal of diatom frustules (Fig. 2c,d; Supplementary Fig. 5). In particular, these holes penetrate through the whole walls of graphene microarchitectures (Fig. 2e), forming aligned hierarchical channels within individual graphene flakes. Obviously, the diatomite-derived graphene powders are distinctly different from RGO and LPEG that are composed of simple planar graphene flakes. Instead, these biomorphic graphene powders are constructed with non-planar microflakes having unique 3D hierarchical channel structures, in addition to the considerably higher crystalline quality.

The surface-group-free nature of such HBG powders was further confirmed by spectroscopic analysis. Fourier transform infrared (FTIR) micro-spectrum of HBG flakes shows no discernible peaks from 600 to 1,800 cm^−1^ (Fig. 2f), in contrast to those of RGO and GO flakes containing hydroxyl (C–OH), epoxide (C–O–C), carbonyl (C=O) and carboxyl (COOH) groups^35^. The absence of these chemical groups reveals that the basal plane of HBG flakes kept intact. Meanwhile, no signal of silicon-oxygen bond (Si–O) was detected in HBG as compared with the FTIR data of biosilica/graphene (before removing the templates), strongly suggesting the complete removal of diatomite templates. X-ray photoelectron spectroscopy (XPS) analysis also demonstrates that HBG flakes are almost free of surface oxygen-containing groups and silicon-containing impurities (Supplementary Fig. 6), consistent with the FTIR and thermogravimetric results (Supplementary Fig. 7). The oxygen percentage in HBG estimated from XPS data is ∼3 wt%, close to the value in graphite (∼2 wt%) but much lower than that of RGO (∼15 wt%) (Supplementary Table 1).

In analogy with previously reported growth of graphene via CVD on planar silica-based substrates^27^,^28^, lattice oxygen at the surface defect sites of amorphous biosilica can also accelerate the capture and pyrolysis of hydrocarbons at high temperature through the formation of C–O and OH bonds. The nucleation and growth process of graphene can thus be promoted with the formation of minimum-energy sp^2^-bonded carbon structure^36^. During the reaction process, the deposition of graphene layers on biosilica surface could be gradually suppressed with increasing layer thickness, due to the attenuated surface activity of substrates coated by continuous graphene layers. This self-limited growth mechanism could be further confirmed by examining the CVD growth of graphene on the surface of individual silica spheres (Supplementary Fig. 8), revealing graphene layer numbers of less than *ca.* 5 even within sufficient growth durations. Therefore, by selecting suitable methane concentrations, the thickness of graphene layers on the 3D biosilica surface can be on-demand controlled, which is different from those of RGO and LPEG materials.

**Figure 3:**
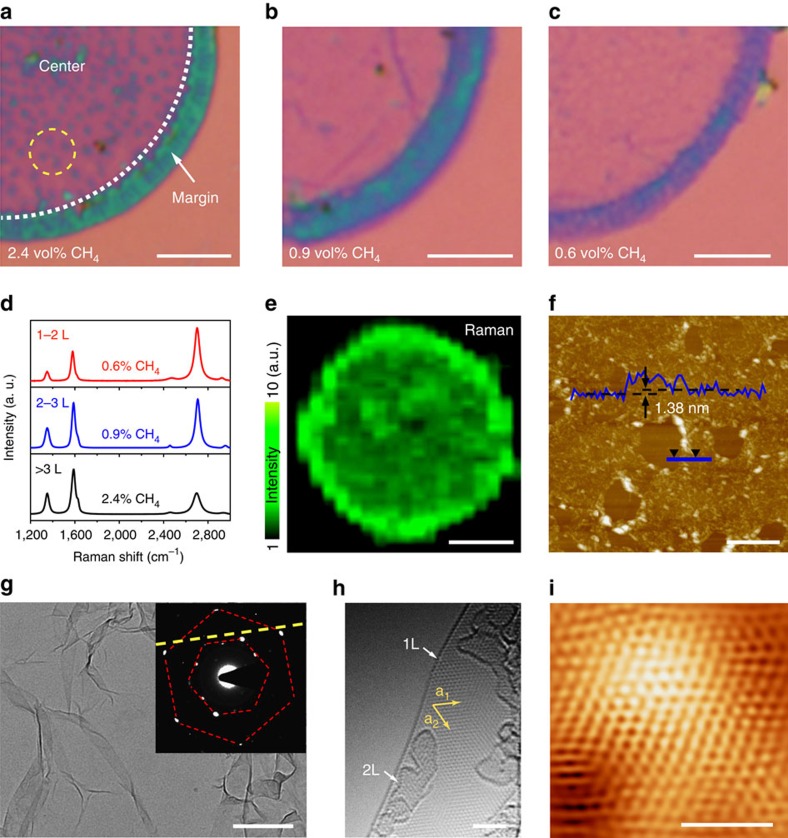
Thickness-controllable growth of high-quality HBG powders. (**a**–**c**) Optical microscope images of HBG flakes grown at a CH_4_ concentration of 2.4, 0.9 and 0.6 vol%, respectively after transferred onto 300 nm SiO_2_/Si substrates. (**d**) Corresponding Raman spectra obtained from **a** to **c**. (**e**) Raman mapping image (2D peak from 2,650 to 2,750 cm^−1^) of HBG flake shown in **a**. (**f**) Atomic force microscope image of the porous graphene flake inheriting from diatom frustule. The height profile taken along the blue line shows a relative height of ∼1.38 nm to background. (**g**) TEM image and selected-area electron diffraction pattern (as an inset) of graphene layers grown at 0.6 vol% CH_4_. (**h**) Low-voltage, aberration-corrected, high-resolution TEM image of graphene layers, exhibiting the six-fold symmetry and edges of single layer and two layers. (**i**) STM image (*I*_t_=1.3 nA, *V*_s_=70 mV) of the graphene honeycomb lattice. Scale bars, 5 μm (**a**–**c**), 10 μm (**e**), 1 μm (**f**), 500 nm (**g**), 2 nm (**h**) and 1.5 nm (**i**).

Figure 3a–c exhibit the optical microscope images of the central and marginal regions of a quarter of HBG flake grown at a CH_4_ concentration of 2.4, 0.9 and 0.6 vol%, respectively, after transferred onto 300 nm SiO_2_/Si substrates. Generally, the colour contrast reflects the thickness of graphene layers, with the darker area assigned to the thicker graphene films, though it is complicated here by the 3D hierarchical structures. Within a single HBG flake, the edge region always shows a darker contrast, which is attributed to the construction of densely aligned graphene channels replicated from the edge channel structures of original diatom frustules. Similar effects can be clearly observed in the central area when increasing the CH_4_ concentration. The dark dots shown in Fig. 3a are corresponding to the graphene channels grown on the side walls of original frustule central pores (Supplementary Fig. 9). The colour contrast seen here arises from the complicated 3D stacking of graphene layers and channels after removal of biosilica template.

The actual layer number of graphene can be further estimated from Raman analyses of 2D/G intensity ratio and full width at half maximum of 2D band. For instance, the HBG flakes corresponding to Fig. 3a–c are mainly composed of few layers, 2–3 layers, and 1–2 layers of graphene, respectively (Fig. 3d and Supplementary Fig. 10). At a low CH_4_ concentration (0.6 vol%), the HBG is mostly monolayer graphene with a 2D/G intensity ratio of *ca.* 1.8 and a 2D band full width at half maximum of 47 cm^−1^, well consistent with the single-layer graphene on planar oxide substrates^27^,^33^,^37^. Note that the layer numbers are only estimated for graphene grown on the flat area of frustule template for simplicity. It can be seen from the Raman mappings shown in Fig. 3e and Supplementary Figs 11 and 12 that uniform graphene growth has been achieved on the biosilica template under varied CH_4_ concentrations. Again, the higher Raman intensity contrast in the 2D and G maps at edges indicates densely aligned graphene channels, in accordance with the optical microscopic images. Shown in Fig. 3f is the atomic force microscope image of an individual HBG flake grown at 0.6 vol% CH_4_ after transferred onto SiO_2_/Si substrates. A statistical average height collected on different graphene flakes is ∼1.5 nm (Supplementary Fig. 13), which could correspond to the overlapped single-layer graphene^37^,^38^. Moreover, TEM measurements (Fig. 3g and Supplementary Fig. 14) also confirm the thin-layer feature and good crystallinity of HBG flakes. Corresponding selected-area electron diffraction pattern (Fig. 3g inset) shows typical six-fold symmetrical spots indexed as the in-plane (hkil) reflections of graphene^39^. In a low-voltage, aberration-corrected, high-resolution TEM image (Fig. 3h), both the edges of one and two graphene layers, and the six-fold symmetrical atomic structures typical for graphene are identified. Meanwhile, the locally convex structures of 3D HBG layers are also clarified (Supplementary Fig. 15). Scanning tunnelling microscopy (STM) images shown in Fig. 3i and Supplementary Fig. 16 again demonstrate the atomic resolution honeycomb lattice for the HBG flake transferred onto a 4H-SiC (0001) substrate.

### Solution processability of HBG flakes

**Figure 4:**
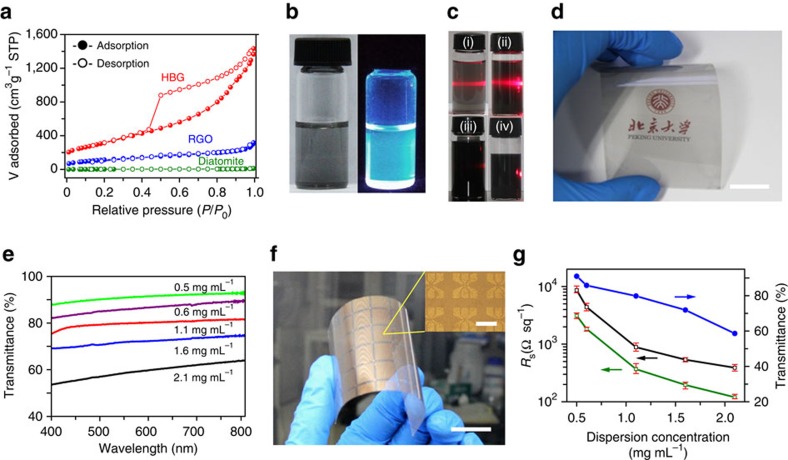
Solution processability of HBG flakes for transparent conducting films. (**a**) Nitrogen adsorption–desorption isotherms of HBG, RGO and diatomite powders. (**b**) Photographs of HBG NMP dispersions at 0.03 mg ml^−1^, showing photoluminescence under ultraviolet light. (**c**) Photographs (i–iv) of EC-stabled HBG in ethanol/terpineol at a concentration of 0.02, 0.05, 0.5 and 4 mg ml^−1^, respectively. (**d**) Photograph of a flexible transparent HBG/mica film (4.5 inch) with 80% transparency. (**e**) Optical transmittances of HBG/mica films prepared at different graphene concentrations. (**f**) Photograph of a patterned graphene Hall bar array fabricated on mica. Inset: optical microscope image of the Hall bar array. (**g**) Sheet resistance (green and black curves correspond to the nitric acid-treated and -untreated films, respectively) and transmittance (blue curve without treatment taken at 550 nm) of graphene films as a function of HBG concentration. The error bars represent the standard deviation of multiple measurement results. Scale bars, 2 cm in (**d**) and (**f**), 200 μm in (**f**) inset.

It is known that good dispersion of graphene powders is the premise for various low-cost and high-performance graphene products such as graphene conductive films, ultralight foams and high-concentration solutions. However, it still remains a challenge to inhibit the restacking of pristine graphene due to its strong interfacial van der Waals interactions and large surface area. As envisioned from the 3D textural features of these atomically thin-layered graphene frameworks, the biomorphic graphene powders show an improved specific surface area compared with the diatomite templates (9.7 m^2^ g^−1^) and the RGO powders (420.9 m^2^ g^−1^), which is calculated to be 1,137.2 m^2^ g^−1^ by the Brunauer–Emmett–Teller surface area analysis (Fig. 4a and Supplementary Table 2). The profound increase (∼116 and 2.7 times of that of diatomite and RGO, respectively) is attributed to the lightweight and non-stacking thin-layer nature of HBG powders. In order to probe the dispersion stability of such 3D graphene powders with a large surface area, they were dispersed into various solvents followed by sedimentation, with the dispersion of RGO powders as a reference (at the same concentration of 0.08 mg ml^−1^; Supplementary Fig. 17). *N*-Methyl-2-pyrrolidone (NMP) was found to be best one among the tested solvents for dispersing graphene layers into a stable suspension, due to its perfect match of the polar–polar affinity with graphene (the negative free energy of graphene/NMP mixing system)^40^,^41^. The synthesized HBG powders can be directly and fast dispersed in NMP without precipitation, the 3D porous non-stacked structures of which provide the space for the effective penetration of NMP solvent. Distinguished from the complete precipitation of RGO sheets, HBG flakes presented relatively good dispersity and stability in NMP after standing for 1 day, which could be attributed to the locally curved structures preserving a weak interlayer interaction. More intriguingly, as shown in Fig. 4b, the transparent HBG dispersion with a concentration of 0.03 mg ml^−1^ exhibits blue photoluminescence, which could be attributed to the strongly localized *π* state of the sp^2^ clusters by the electric states of sp^3^ defect matrix^42^. This blue photoluminescence should serve as a direct evidence for the good solubility of HBG flakes in NMP, which is similar to that of nanocarbons (for example, carbon dots and carbon nanotubes), but distinguished from that of carbon powders presenting poor solubility in NMP^43^.

Furthermore, HBG dispersions with varied concentrations from 0.03 to 0.11 mg ml^−1^ were characterized by Zeta-potential analyses, and compared with RGO dispersions (Supplementary Fig. 18). The decreases of solubility for HBG and RGO in NMP are revealed with the increase of dispersion concentrations, respectively, due to the gradual saturation of graphene dispersions. Nevertheless, it also achieves a comparable production level to the shear-exfoliated process of graphite^12^, which is operated to obtain monolayer and few-layer graphene in NMP with flake sizes of <1 μm (at the concentration up to 0.07 mg ml^−1^). Besides, a time-consuming chemical/physical exfoliation process (from several hours to several days) is also required to yield monolayer or few-layer graphene sheets for most RGO and LPEG dispersions, in contrast to the fast wet-etching process for HBG powders. Alternatively, high-concentration dispersion (>1 mg ml^−1^) is also achieved for ethyl cellulose(EC)-stabilized HBG flakes in ethanol/terpineol within a short timescale of about several minutes, which also shows an excellent stability (Supplementary Fig. 19). The homogeneous graphene ethanol/terpineol dispersions are prepared with variable concentrations from 0.02 to 4 mg ml^−1^, as evidenced by their uniform colour contrasts and Tyndall effect (Fig. 4c).

### Graphene transparent conductive films

For transparent electrode applications, the HBG dispersions can be utilized to prepare large-area graphene conductive films on flexible substrates at room temperature. A wire-wound rod-coating technique was applied to these HBG dispersions to prepare a flexible, continuous and thickness-controllable graphene films on transparent mica substrates (Fig. 4d and Supplementary Figs 20 and 21). An empirical concentration of at least 0.5 mg ml^−1^ for HBG dispersions was found to be essential for coating continuous and uniform graphene films. The optical transmittance of graphene films decreases with the increase of film thickness (Fig. 4e). Four-probe resistance measurements were performed to evaluate the conductive properties of graphene/mica films (Fig. 4f), and the sheet resistances and corresponding transmittances of various graphene films are plotted in Fig. 4g, respectively. Together with the increase of HBG concentration, the sheet resistance of graphene film drops quickly from ∼8.6 to ∼0.39 kΩ sq^−1^ (corresponding conductivity change from ∼10,400 to ∼55,000 S m^−1^) while the optical transmittance decreases from 91 to 58% at 550 nm. To enhance their conductivity, these graphene films are further immersed into nitric acid (HNO_3_). After HNO_3_ treatment, the average sheet resistance of graphene films drops to 0.36 kΩ sq^−1^ at a transmittance of ∼80% at 550 nm (see resistance statistics in Supplementary Fig. 22). And the corresponding conductivity is calculated to be ∼110,700 S m^−1^, which is at least one order of magnitude higher than the previously reported hydrazine-reduced RGO (<1,000 S m^−1^ at 80% transmittance) and LPEG (∼6,500 S m^−1^ at 43% transmittance) counterparts^5^,^6^,^40^,^44^,^45^ (Supplementary Table 3). This marked increase could be attributed to the removal of residual absorbed organics such as EC molecules^46^, as well as the slight charge doping^9^, which are efficient for enhancing the conductivity of graphene flakes. Further systematic comparisons given in Supplementary Fig. 23 demonstrate the superior electrical conductivities of these graphene films with regard to various previously reported solution-processed graphene films in a broad range of optical transmittances. This can be attributed to the better crystallinity and optical semi-transparency of 3D porous graphene building blocks. Besides, HBG powders also exhibit much higher conductivity and better dispersity than those of previously reported graphitic carbon powders (Supplementary Table 4), which offer very high potential for exploiting new graphene-based materials towards energetic and environmental applications.

**Figure 5:**
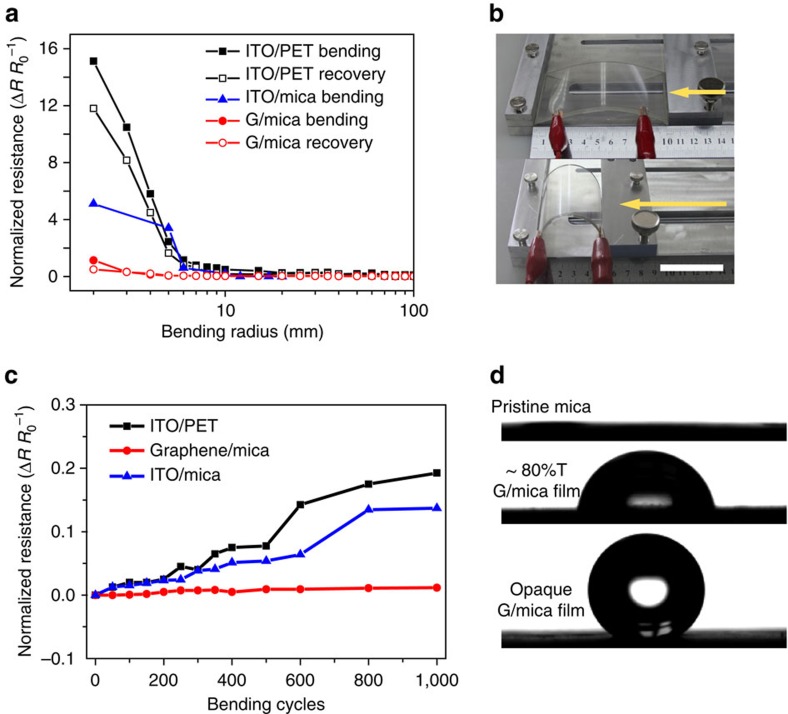
Mechanical and hydrophobic properties of graphene/mica films. (**a**) Normalized resistance of HBG/mica (∼50 μm thickness) compared with commercial ITO/PET and sputtered ITO/mica for different bending radii. (**b**) Bending process. Scale bar, 5 cm. (**c**) Resistance change of HBG/mica (∼50 μm thickness) over bending cycles. The data of sputtered ITO/mica in **a** and **c** were collected and calculated from ref. 47. (**d**) Water contact angles of pristine mica (0°), HBG (80% transparency)/mica (81.6°) and HBG/mica thick film (142.3°), respectively.

The electrical resistance over bending was further monitored to evaluate the mechanical durability of HBG/mica film (Fig. 5a,b). In one bending cycle, the resistance showed a slight increase at a bending radius of 2 mm, which was recovered after release. In contrast, the commercial ITO/poly(ethylene terephthalate) (PET) film (0.2 μm, ∼400 Ω) and the sputtered ITO/mica film^47^ exhibited marked resistance increases at a bending radius of 6 mm without recovery after release. Surprisingly, the HBG/mica film showed no obvious increase (<3%) of electrical resistance over 1,000 bending cycles as seen in Fig. 5c, demonstrating its excellent bending durability as opposed to ITO/PET and sputtered ITO/mica films. The excellent bending tolerance of HBG/mica film over fragile ITO arises from the efficient overlapping and electrical contact between large HBG flakes, which offers a great freedom for fabricating graphene-based wearable electronic devices. Furthermore, the HBG/mica surface is hydrophobic, having a water contact angle of 81.6° with a ∼80% transmittance of graphene film (Fig. 5d). For a thicker graphene drop-cast film on mica (∼2 μm in thickness), the water contact angle further increased up to 142.3°. Therefore, the surface wettability of HBG/mica can be on-demand modulated simply by controlling the HBG film thickness, providing the possibility for fabricating anti-water mica plates.

### Flexible printed graphene electrodes

**Figure 6:**
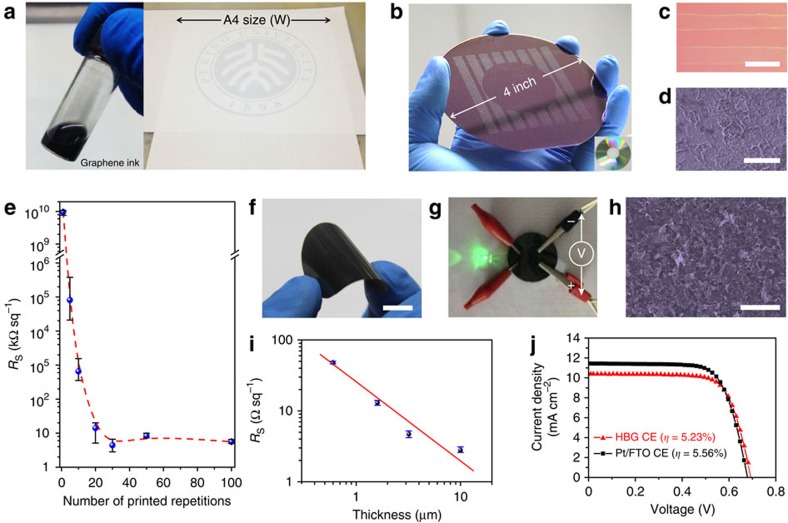
Flexible printed graphene electrodes. (**a**,**b**) Photograph of inkjet-printed graphene on A4-sized paper (**a**) and 4-inch SiO_2_/Si wafer (**b**), respectively. (**c**,**d**) Optical microscope (**c**) and false colour SEM (**d**) images of printed graphene strips on SiO_2_/Si substrates after 10 printing repetitions. (**e**) Sheet resistance of printed graphene strips as a function of printing repetitions. (**f**) Photograph of a vacuum-filtered graphene film hot-pressed by roll-to-roll printing technique on a PET substrate. (**g**) A green LED lighted through a graphene/PET film at a starting voltage of 2 V. (**h**) False colour SEM image of the graphene/PET film. (**i**) Sheet resistance versus thickness for the graphene film on PET substrate. (**j**) *J–V* characteristics for the dye-sensitized solar cells with a Pt/FTO counter electrode and a graphene counter electrode, respectively. The error bars represent the standard deviation of multiple measurement results. Scale bars, 200 μm (**c**), 5 μm (**d**), 1 cm (**f**) and 10 μm (**h**).

Inkjet printing is an attractive approach for fabricating patterned graphene electrodes. As demonstrated in Fig. 6a,b, the graphene ink with a volume ratio of 1:4 (Supplementary Fig. 24, viscosity: ∼12 mPa·s; surface tension: ∼28 mN m^−1^, 25 °C) could be directly written on paper and SiO_2_/Si wafer, well comparable with the commercial inks. The optical and SEM images in Fig. 6c,d of printed stripes on SiO_2_/Si substrate exhibit stripes of ∼100 μm width with uniform distribution of graphene flakes, where the so-called ‘coffee ring' effect is almost not found. The sheet resistance of thus-printed graphene film reaches a relatively low value of ∼5 kΩ sq^−1^ after 30 printing repetitions with this graphene ink (Fig. 6e), achieving a similar level to that previously reported for the inkjet-printed LPEG^48^,^49^. Further decrease of sheet resistance would be possible for less printing passes with further concentrated graphene inks. A roll-to-roll transferred printing technique was also employed to make graphene thick films hot-pressed on ethylene-vinyl acetate-coated PET substrates for flexible electrodes (Fig. 6f–h and Supplementary Fig. 25). In this case, the graphene film shows an electrical conductivity as high as 66,500 S m^−1^ (Fig. 6i; Supplementary Table 3), which is superior to its RGO and LPEG counterparts^6^,^7^,^12^,^50^. In particular, thus-obtained graphene film on PET worked well as the counter electrode of the dye-sensitized solar cells (DSSCs). A power conversion efficiency of ∼5.23% (Fig. 6j) was achieved, competitive with efficiency levels of Pt/FTO-based DSSCs.

## Discussion

In summary, we have developed a scalable and surface-oxygen-assisted CVD route for producing a type of 3D biomorphic graphene powder materials. This approach utilizes the naturally abundant and industrially widely used diatomite as the growth template, with the surface growth mediated by a self-limited mechanism. Different from the chemically exfoliated graphene powders, the derived HBG materials are highly crystallized with layer-thickness controllability and less noncarbon impurities. In particular, the individual graphene flakes preserve the 3D hierarchical structures of original diatom frustules, effectively overcoming the interlayer stacking and hence giving a wonderful solution dispersion performance essential for various solution-based processes. The graphene films made from these biomorphic graphene flakes exhibit much higher conductivity and excellent bending tolerance than the reported RGO and LPEG counterparts, which is expected to find wide applications in flexible transparent electrodes, polymer composites, conducting inks and printed electronics. The DSSCs using such flexible electrodes also have demonstrated a comparable level of power conversion efficiency with the expensive platinum-based counterparts.

On the laboratory scale, these purified HBG powders have an estimated yield of *ca.* 1% (the g/g weight ratio of graphene/diatomite), presenting a comparable production rate to those of RGO and LPEG powders (Supplementary Table 5). In view of the abundance and low cost of diatom frustules, the bio-inspired CVD process is amenable to scale-up to an industrial level. More importantly, as a special form of graphene materials, 3D biomorphic graphene microarchitectures could pave the way for developing novel topologically structured carbon materials for energetic and environmental applications. Not limited to graphene powders, the presented CVD route could also be extended to the 3D scalable growth of other atomically thin-layered materials such as two-dimensional MoS_2_ and *h*-BN, with the merits of mass production, high crystalline quality and low cost.

## Methods

### Purification of diatomite

The diatom frustules were purified from raw diatomite to obtain the ultrapure biosilica microflakes following several steps. First, the as-received diatomite powders (Aldrich) were immersed and stirred overnight in nitric acid (2 mol l^−1^) and sulphuric acid (1 mol l^−1^) to remove the organic and metal impurities, respectively. After the recycled filtration and the deionized water cleaning, the biosilica microflakes with different particle sizes were separated from the filtrated diatomite using recycled sedimentation processes in acetone.

### Growth of hierarchical biomorphic graphene on diatomite

Diatomite powders were put into a three-zone tube furnace. The tube was pumped to vacuum and rinsed with 300 standard-state cubic centimetre per minute (sccm) of 10 vol% hydrogen-mixed argon gas to remove air before it was heated to 1,000 °C within 40 min at ambient pressure. The samples were then held at this temperature for 100 min under a constant mixed gas flow of H_2_/Ar carrier gas (300 sccm) and methane (2 sccm) for graphene growth. The thickness of graphene layers could be controlled by varying the concentration of methane gas. After the furnace was cooled to room temperature, light grey powder was obtained. The powder was then immersed in a hydrogen fluoride (HF) etching solution (molar ratio of HF: H_2_O: EtOH is 6.7: 27.8: 5.1) at room temperature (or a 6 mol l^−1^ NaOH solution at 80 °C) overnight to remove the biosilica templates. After complete rinsing with water and ethanol, the graphene powder was obtained by freeze-drying at −90 °C and reduced pressure (∼1 Pa) for 24 h. For comparison, RGO was reduced from GO with hydrazine hydrate by modified Hummer's method^51^.

### Preparation of flexible graphene films

An EC-stabilized graphene dispersion was obtained by adding the as-grown graphene powder into ethanol/terpineol solution containing EC (48% ethoxyl, Aldrich) according to Hersam's method^52^. A 0.001 g ml^−1^ solution of EC in ethanol was first mixed with the graphene powders. After separated by filtration, the graphene/EC sediment was redispersed in ethanol/terpineol solution with volume ratio of 9: 1 by shaking up to form a stable graphene suspension with different concentrations. One millilitre of the graphene dispersions was dropped on the edge of a mica substrate (50 μm in thickness). A Meyer rod (18#, RD Specialties, USA) was pulled over the solution to leave a uniform wet film. During the natural drying process, the graphene films were pressed under a 1 kg weight overnight to improve their formability. After drying, the graphene films were heated in Ar/H_2_ atmosphere at 350 °C for 60 min, and then rinsed with acetone and dried by blowing with nitrogen. The thickness of graphene films after drying (*H*_dry_) can be controlled by adjusting the concentration of HBG dispersion, according to the following equation^53^, *H*_dry_*=H*_wet_ *C*_o_ *ρ*^−1^, where the thickness of graphene wet films (*H*_wet_) can be regarded as 10% of the wire diameter of the used wire-wound rod, *C*_o_ is the concentration of the graphene dispersion, and *ρ* is approximated as 1.8 g cm^−3^ referring to the density of graphene oxide films^44^, since HBG layers could not be as closely packed as graphite. For the HBG concentrations of 0.5, 0.6, 1.1, 1.6 and 2.1 mg ml^−1^, the thicknesses of dried graphene films can be calculated to be about 11.1, 13.3, 25.1, 35.6 and 46.8 nm, respectively.

Prior to inkjet printing, the solution of graphene/EC in ethanol/terpineol was bath ultrasonicated for 60 min to yield small graphene fragments, and then filtered through a polycarbonate membrane with 1 μm pores (25 mm in diameter, Millipore). The filtered solution was concentrated into graphene/EC terpineol dispersion by heating at 120 °C to remove ethanol, yielding a graphene ink at a concentration of ∼0.2 w/v%. The essential requirement of graphene ink compatible with inkjet printing is its viscosity and surface tension. For successful inkjet printing, the graphene terpineol dispersion was diluted with ethanol at different volume ratios ranged from 1:8 to 1:2 to tailor the viscosity and surface tension. An Epson R330 inkjet printer with a T0851 cartridge was used together with graphene ink for printing pictures on A4 paper and narrow lines on SiO_2_/Si wafers at a substrate temperature of 100 °C.

The graphene films could be also prepared by vacuum filtration, followed by a roll-to-roll transferred printing process on PET substrate. A volume of 200 ml of graphene ethanol dispersion with a concentration of 40 μg ml^−1^ was vacuum-filtrated using a polycarbonate membrane with 0.45 μm pores (47 mm in diameter, Millipore). The naturally dried graphene film was then pressed on the ethylene-vinyl acetate-coated PET substrate with the graphene side in contact with the substrate at *ca.* 120 °C.

### Characterization

Optical microscope (Olympus DX51 microscope), atomic force microscope (Vecco Nanoscope IIIa), SEM (Hitachi S-4800; acceleration voltage 5–30 kV), TEM (FEI Tecnai F20; acceleration voltage 200 kV), aberration-corrected atomic-resolution TEM (FEI Titan 300–80; 80 kV), Raman spectroscopy (Jobin Yvon LabRAM HR 800UV; 514.5 nm, 25 mW), ultrahigh vacuum-variable temperature-STM (Omicron, constant current mode), FTIR (Thermo Scientific, Nicolet iN10 MX, transmission mode), XPS (Kratos Analytical Axis-Ultra spectrometer; Al K_α_ X-Ray source), thermogravimetric (SDT Q600), XRD (Phillips X' XPert Pro MPD; Cu K_α1_ *λ*=1.540598 Å, 40 kV, 100 mA), ultraviolet-vis spectroscopy (Perkin Elmer Lambda 950) and static contact-angle measurements (OCA-20 contact angle system) were conducted to characterize the materials, respectively. For SEM and TEM characterization, a PMMA-assisted transfer process was taken for samples onto a SiO_2_/Si substrate (300 nm oxide layer) and a lacey carbon film supported on copper grids, respectively. For STM characterization, the 4H-SiC(0001) substrates were pre-annealed at *ca.* 900 °C in vacuum to improve conductivity.

### Device fabrication and measurements

The use of dielectric mica substrates facilitated the transfer-free batch fabrication of graphene transparent electrodes, which is also compatible with high frequency mica capacitors. Standard photolithography was used to define the selected region and fabricate arrays of Hall bar devices. Plasma etching (50 W, 60 s) was used to remove the unwanted regions. Thermal evaporation (UNIVEX 300, Leybold Vacuum) was performed to deposit the metal electrodes with chromium/gold (5 nm/100 nm) as the contacts, which were found to form ohmic contact through annealing at 350 °C in Ar/H_2_ atmosphere. Four-probe electrical measurements were carried out in a Micromanipulator 6200 probe station with a Keithley 4200 semiconductor characterization system.

A sandwich-type solar cell was assembled by placing a PET-supported graphene film (counter electrode) on a N719 dye (Solaronix)-sensitized TiO_2_ photoelectrode on FTO glass (working electrode, effective area of 0.3 × 0.3 cm^2^), and clipped together as an open cell for measurements. The cell was then filled with a liquid electrolyte composed of 0.1 M anhydrous LiClO_4_, 0.12 M I_2_, 1.0 M DMPII and 0.5 M tert-butylpyridine in dehydrated acetonitrile by capillary force. For comparison, a standard platinum-based counter electrode was also used. The photocurrent–voltage (*J–V*) measurements were taken using a digital source meter (Keithley 2,400) and a solar simulator (Newport, Oriel class A, SP91160A, USA) under AM 1.5 G spectra with a light power density of 100 mW cm^−2^.

### Data availability

The data that support the findings of this study are available from the corresponding author upon request.

## Additional information

**How to cite this article:** Chen, K. *et al*. Growing three-dimensional biomorphic graphene powders using naturally abundant diatomite templates towards high solution processability. *Nat. Commun.* **7,** 13440 doi: 10.1038/ncomms13440 (2016).

**Publisher's note:** Springer Nature remains neutral with regard to jurisdictional claims in published maps and institutional affiliations.

## Supplementary Material

Supplementary Information Supplementary Figures 1-25, Supplementary Tables 1-5 and Supplementary References
