# Supplementary material for: Growing three-dimensional biomorphic graphene powders using naturally abundant diatomite templates towards high solution processability
Source: Nat Commun. 2016 Nov 7;0:13440. doi: 10.1038/ncomms13440 (PMC5103074; doi:10.1038/ncomms13440)
Supplement: Supplementary Information — Supplementary Figures 1-25, Supplementary Tables 1-5 and Supplementary References [file ncomms13440-s1.pdf]

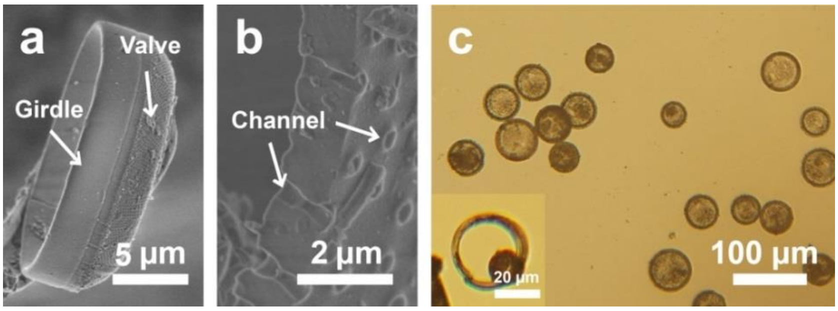

Supplementary Figure 1 | Micromorphology of diatom frustules. a, SEM image of a valve of diatom frustules wrapped by a girdle. **b**, SEM image of cross-section of a porous silica layer of valve. **c,** Optical micrograph of individual valves and girdle (inset) on silicon wafers**.**

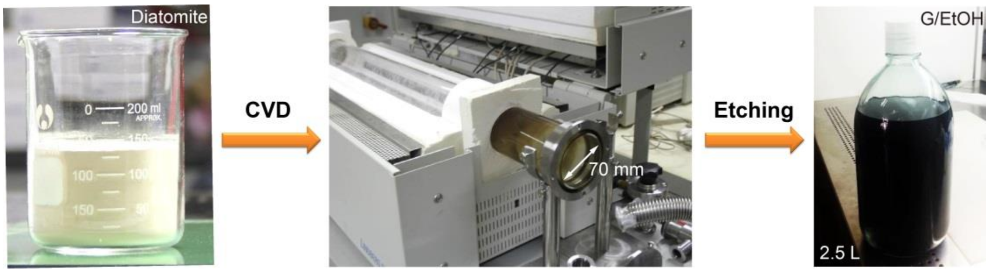

Supplementary Figure 2 | Scalable production process of HBG. The loose bulk density of diatomite is ~0.31 g cm^{-3}.

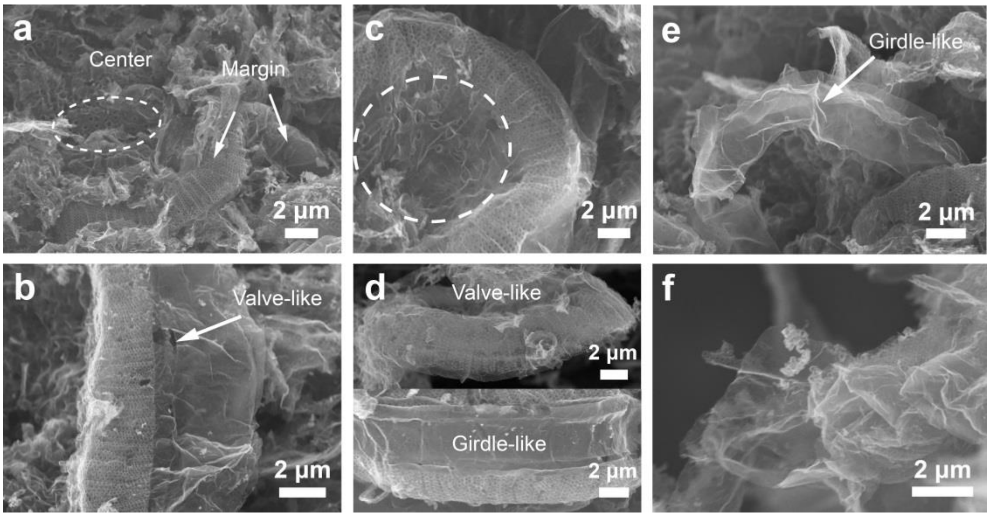

Supplementary Figure 3 | SEM images of graphene flakes with hierarchical biomorphic structures. a, The graphene powder. **b-d,** The valve-like graphene flakes. **e,** A girdle-like graphene flake. **f,** The thin-layer edges of graphene.

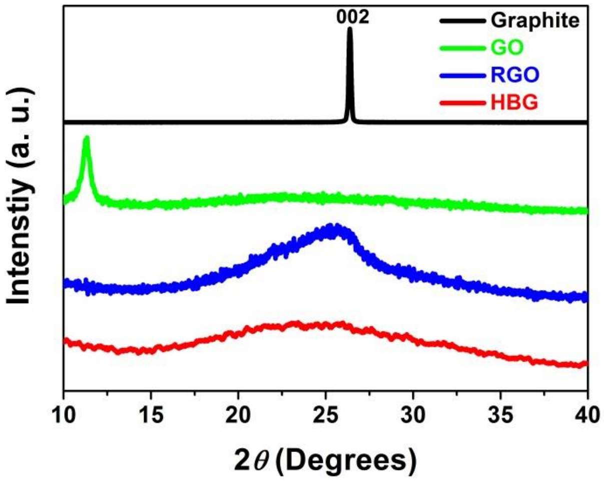

Supplementary Figure 4 | XRD patterns of HBG together with graphite, RGO and GO powders. Powder X-ray diffraction patterns of HBG powders show no obvious peaks from 10° to 40°, indicative of its lack of layered periodic structures of graphite. In contrast, the graphitic carbon powders, together with GO and RGO powders show the obvious (002) X-ray diffraction peaks representing multilayer nature of graphite at about 26°.

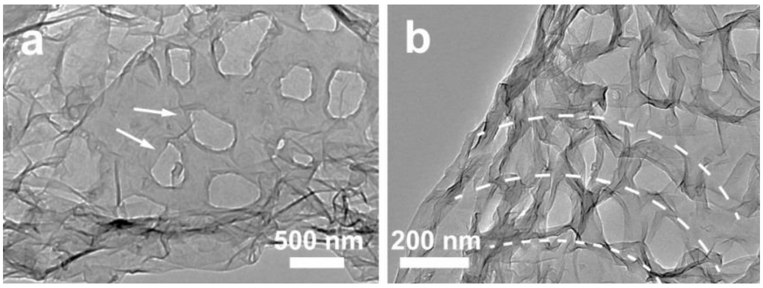

Supplementary Figure 5 | Micromorphology of frustules-derived graphene sheets. TEM images of graphene sheets derived from central (**a**) and marginal (**b**) regions of frustules. The graphene sheets show the loosely arranged bigger pores and the quasi-periodically arranged smaller pores, respectively, in line with the observation of SEM images.

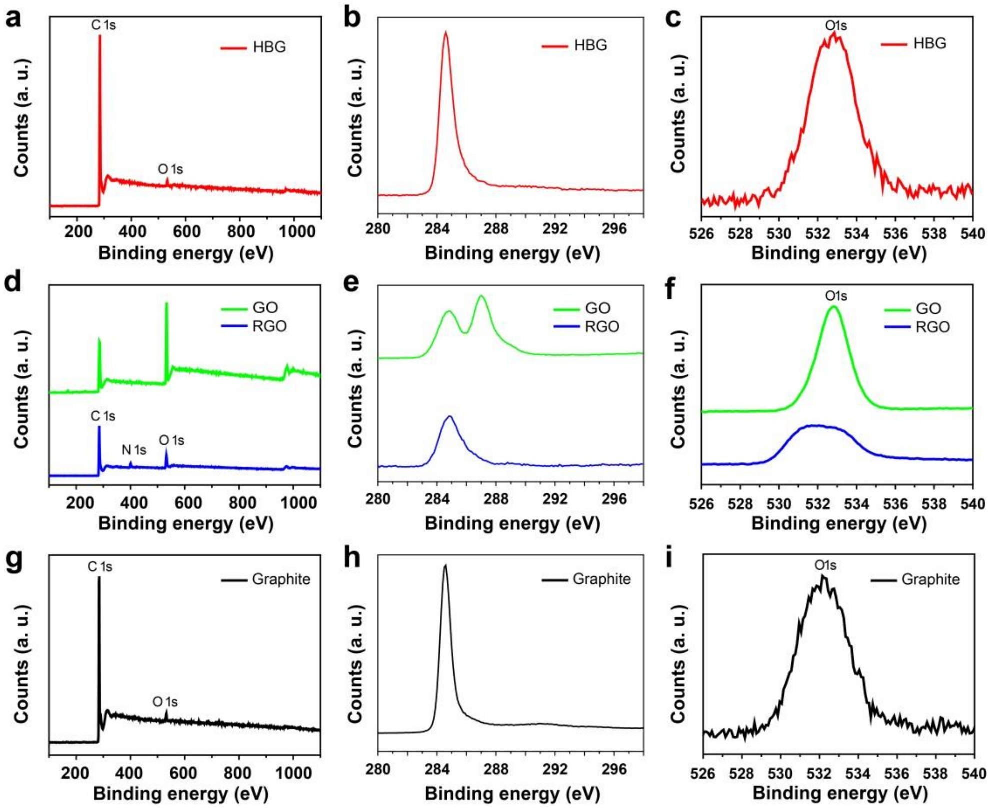

Supplementary Figure 6 | XPS analyses of HBG together with graphite, RGO and GO powders. Survey, C 1s, and O 1s spectra of the HBG (**a, b, c**) together with the graphene oxide (GO) and reduced graphene oxide (RGO) (**d, e, f**), as well as pristine graphite (**g, h, i**). The C1s spectrum of HBG flakes shows an asymmetric peak with small tails expanding to the higher binding-energy region, characteristic of aromatic C=C bonds, which is almost the same as that of natural graphite, but is significantly different from that of RGO. The O 1s spectrum of HBG also shows very weak C-OH peaks at 533.1 eV and minor H-O-H peak at 535 eV (b) for physically absorbed moisture. In contrast, the O 1s spectrum (f) of GO has a single major O 1s peak at 533.1 eV for C-OH. The O 1s spectrum (f) of RGO has several broad O 1s peaks near 532 eV for the residual oxygen-containing groups. The pristine graphite shows a minor O 1s peak at 532.1 eV (d), which is mostly related to physically adsorbed oxygen/moisture for C-OH.

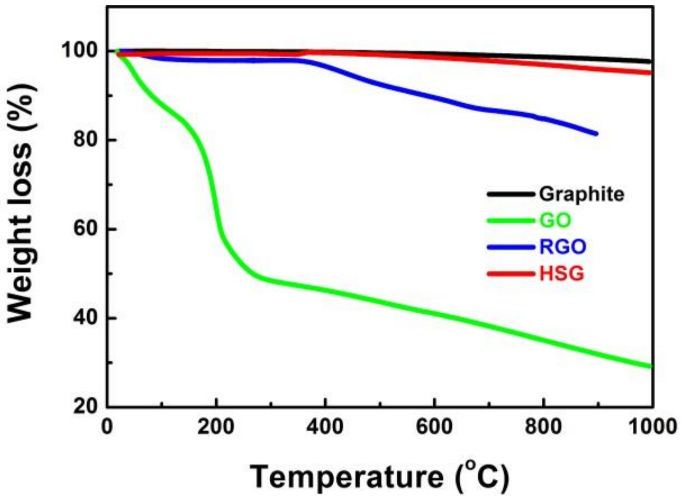

Supplementary Figure 7 | TG analyses of HBG, RGO, GO, and graphite powders. Thermogravimetric analysis (TGA) under N_2 atmosphere is executed to show much higher thermal stability of HBG (less 1% weight loss, the similar as that of graphite) than that of GO and RGO, again indicative of the perfect *sp*^{2} bonded or free-of-functional-groups nature of CVD samples. In contrast, the GO powder demonstrates the obvious weight losses of interlamellar water and its surface oxygen-containing groups. After reduction, the RGO powder also shows a decrease of weight, suggestive of the existence of its residual surface groups.

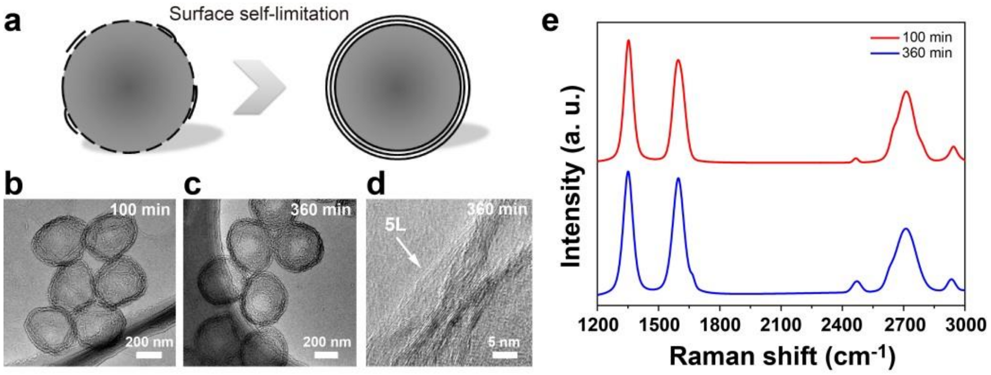

Supplementary Figure 8 | Self-limited growth of graphene layers on the surface of silica spheres with ~400nm diameters. a, Schematic of self-limited growth process. **b, c,** TEM images of graphene layers grown for 100 and 360 min. **d,** HRTEM images of graphene layers grown for 360 min. **e,** Raman spectra of graphene layers grown for 100 and 360 min. In order to further understand the self-limited growth mechanism, we examined the CVD growth of graphene on the surface of individual silica spheres (~400 nm in diameter). At constant growth temperature (1000 °C) and methane concentration (2.4 vol%), the layer numbers of graphene coated on amorphous silica spheres were limited to be less than *ca.* 5, even if the growth time was prolonged from 100 to 360 min. Meanwhile, the corresponding Raman signals of graphene layers did not alter obviously.

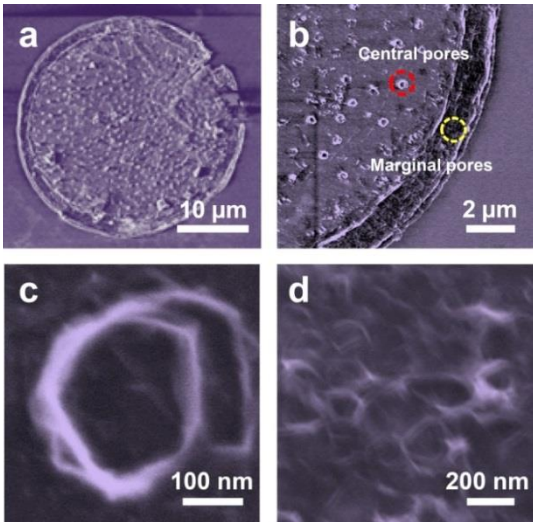

Supplementary Figure 9 | Morphologies of a HBG flake (grown at CH_4 concentration of vol2.4%) transferred onto SiO_2/Si substrates. a, b, False color SEM images of a whole graphene flake (a) and its parts (b). **c, d,** SEM images of central (c) and marginal (d) pores of the graphene flake.

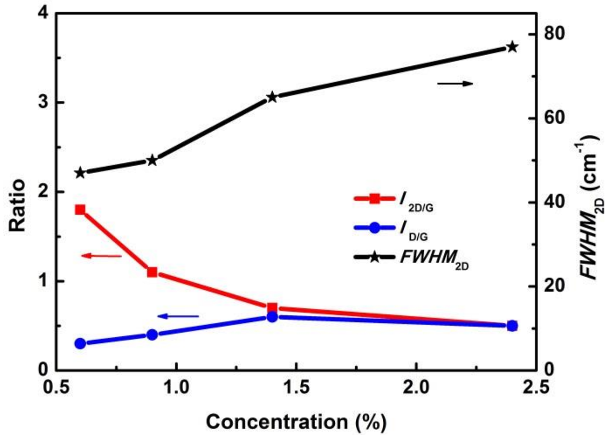

Supplementary Figure 10 | Statistic 2D/G, D/G ratios and FWHMs of HBG versus the CH_4 concentration. It clearly shows the obvious increase in I_{2D}/I_G ratios as the decrease of CH_4 concentrations, suggestive of the thickness decrease of as-grown graphene layers.

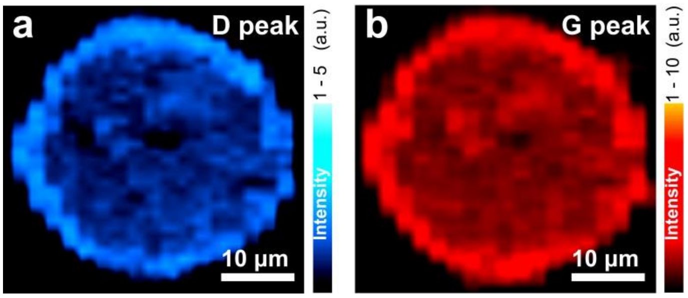

Supplementary Figure 11 | Raman mapping images of the HBG flake. a, D peak. **b**, G peak. Extracted from the integrated intensities of the D, and G peaks, respectively, Raman mapping images show the similar shape to the corresponding SEM image.

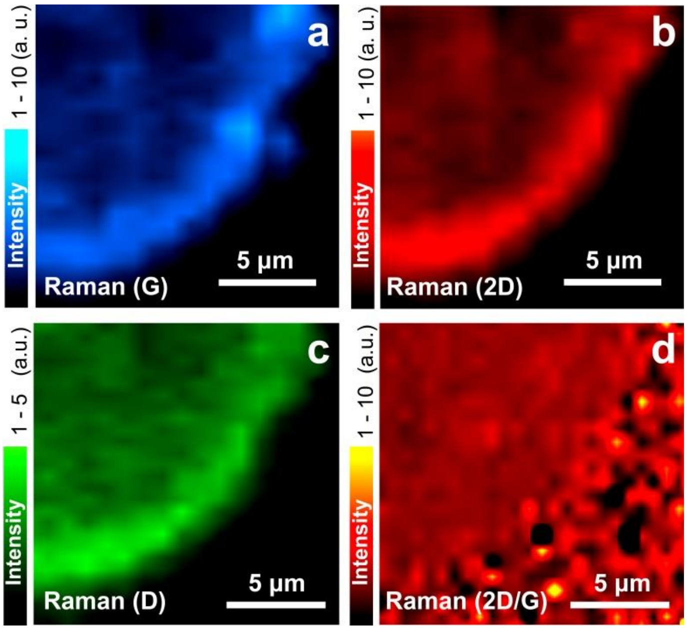

Supplementary Figure 12 | Raman mapping images of the HBG flake grown at CH_4 concentration of 0.6 vol%. (a) G peak; (b) 2D peak; (c) D peak; (d) 2D/G ratio.

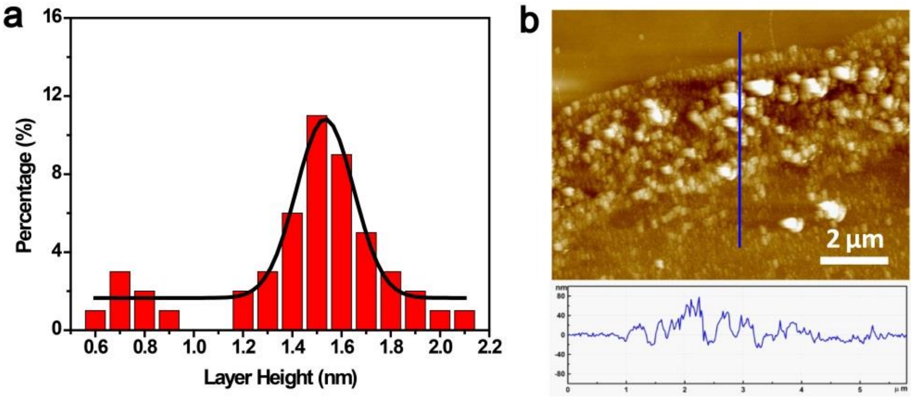

Supplementary Figure 13 | AFM analyses of HBG. a, Height statistic of 50 individual sheets measured, yielding an average height of ~1.5 nm. **b**, AFM image of marginal regions of HBG flakes. Being consistent with the SEM results, AFM image shows a dense arrangement of 3D pores at the margins, which are left after the removal of biosilica substrates.

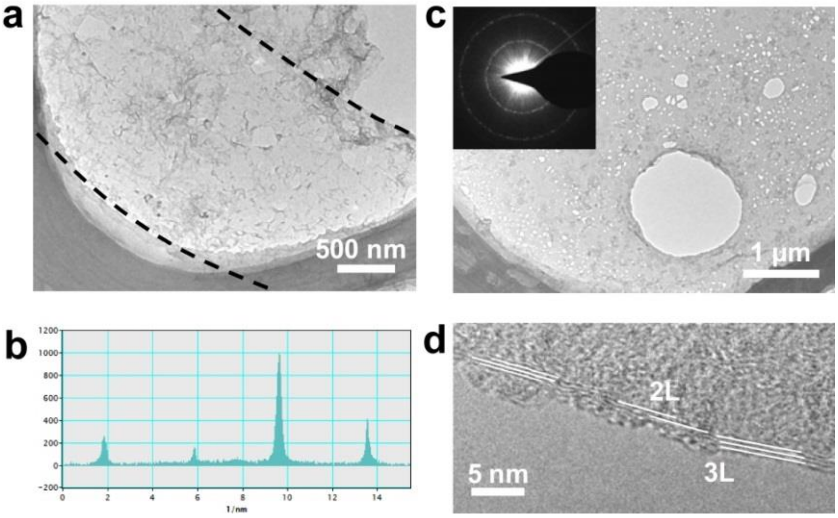

Supplementary Figure 14 | Microstructures of HBG flakes. a, TEM image of the marginal regions of HBG at the CH_4 concentration of 0.6 vol%. **b**, Intensity line profiles of the corresponding SAED patterns. The relative intensities of all inner and outer circle spots are found to be different, which could be contributed to irregular diffractions representing the ripple and wrinkle structures of the suspending non-planar graphene. **c, d,** TEM images of HBG at the CH_4 concentration of 1.6 vol%. The morphologies for 2-3L graphene are also probed. The presence of concentric Debye hexagon rings in SAED pattern (inset) could be attributed to the interconnected or stacked graphene domains.

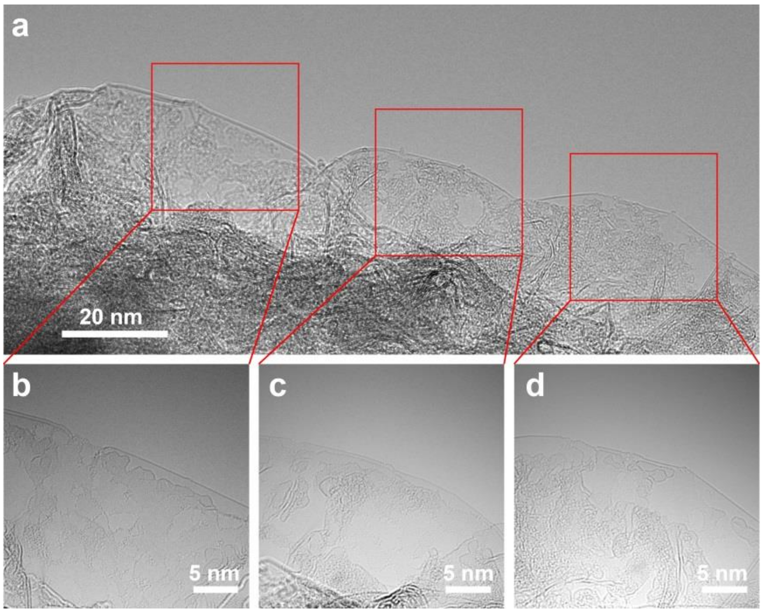

Supplementary Figure 15 | LVAC-HRTEM images of HBG layers with locally convex structures grown at the CH_4 concentration of 0.6 vol%.

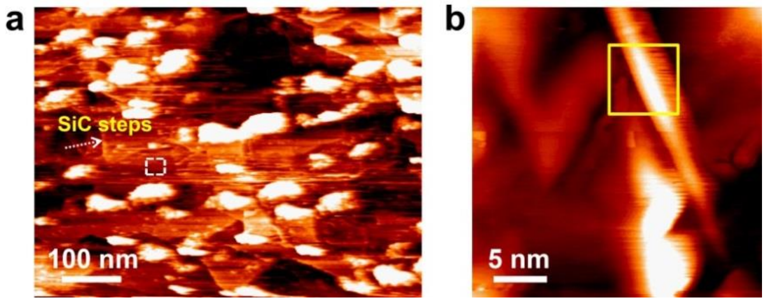

Supplementary Figure 16 | STM images of graphene flakes drop-coated on vacuum-calcinated 4H SiC (0001) substrates.

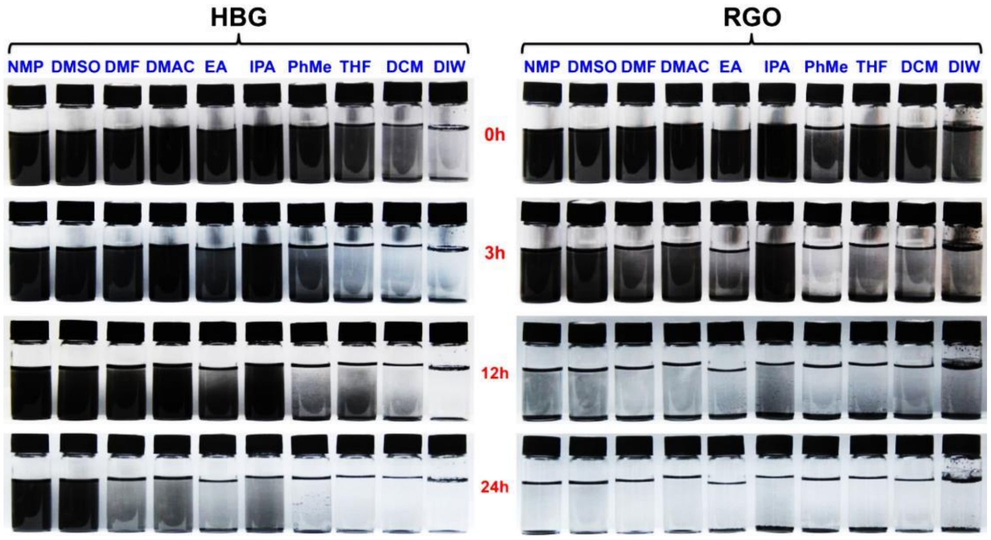

Supplementary Figure 17 | HBG compared with RGO in various solvents for dispersity measurements. (From left to right: N-Methyl-2-pyrrolidinone, dimethyl sulfoxide, N, N-Dimethylformamide, N, N- Dimethylacetamide, ethanol, isopropanol, toluene, tetrahydrofuran, dichloromethane, and deionized water; From top to down: as soon as dispersed, after 3h, after 12h, and after 24h). As soon as HBG and RGO dispersed, a relatively uniform dispersion could be achieved in various polar and non-polar solvents except the deionized water. After 3h, some RGO dispersions began to show an obvious precipitation, such as the RGO toluene, THF, DCM and EA solutions. After 12h, HBG flakes in NMP, DMSO, DMF, DMAc and IPA solvents still preserve a relatively uniform dispersion, while most of RGO dispersions show the obvious sediments. After 24h, compared with almost complete sedimentation of RGO sheets, the relatively good dispersity and stability of HBG flakes have been preserved, which could be attributed to their non-flat curved structures leading to a weak interlayer interaction.

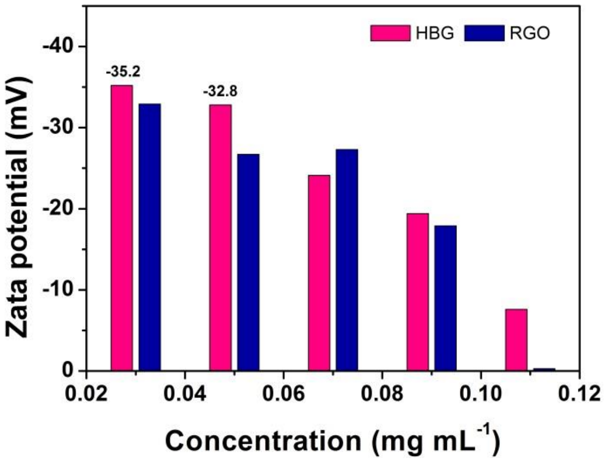

Supplementary Figure 18 | Zeta potentials of HBG and RGO dispersions at different concentrations. The values of Zeta potential were determined using a Zeta-potential analyzer (ZetaPALS, Brookhaven Instruments). Both HBG and RGO dispersions display a good stability, which have the similar Zeta-potential values of -35.2 and -32.9 mV at the concentration of 0.03 mg mL^{-1}, respectively. However, the values of Zeta potential for HBG and RGO dispersions decrease with the increase of the concentration (from 0.05 to 0.11 mg mL^{-1}), suggesting the degradation of graphene solubility in NMP. At rather high concentrations, the increasing collision probability of graphene flakes in NMP leads to the formation of restacked aggregations due to van der Waals interactions and π-π stacking. Nevertheless, all HBG dispersions exhibit higher or comparable Zeta potential than that of RGO dispersions, presenting a relatively better stability.

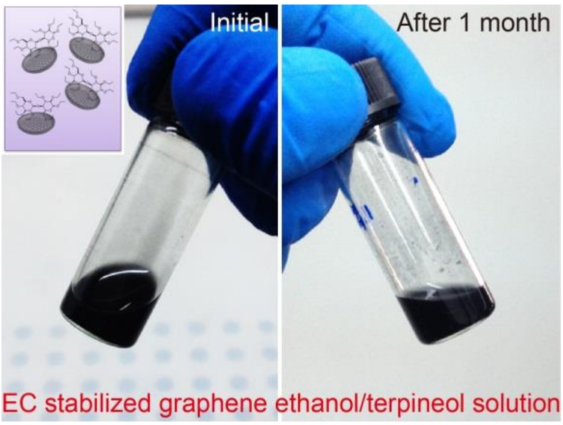

Supplementary Figure 19 | Stability of EC-modified HBG in ethanol/terpinenol. Concentration: ~4 mg ml^{-1}.

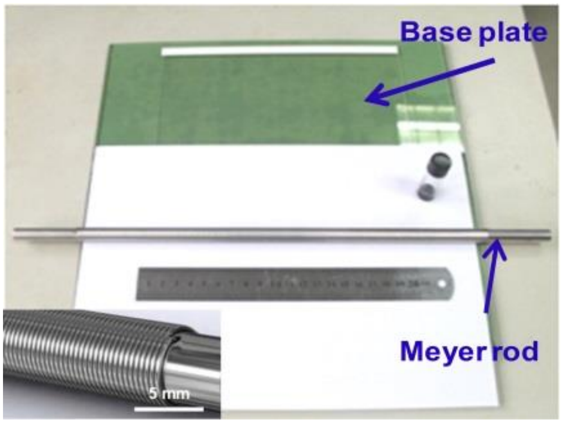

Supplementary Figure 20 | Rod-coating technique of graphene films. a, Photograph of a wire-wound-rod (Meyer rod) setup. **b,** Zoom-in photograph of a Meyer rod.

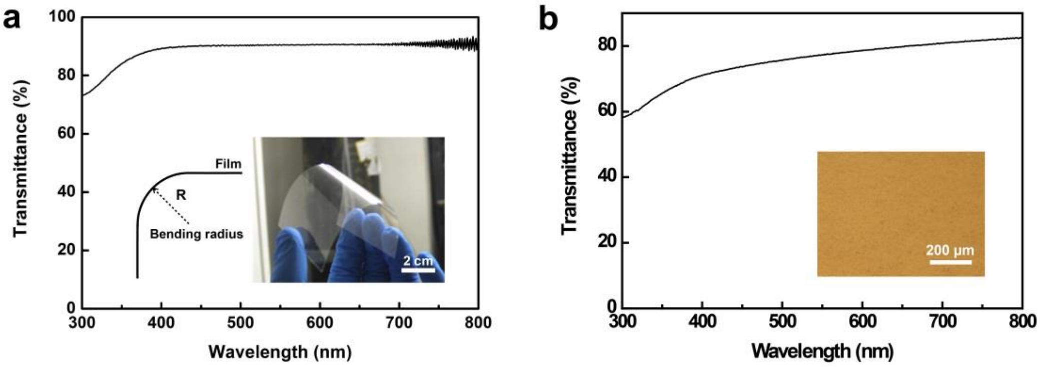

Supplementary Figure 21 | Transparency of flexible mica substrates and graphene/mica films. a, Transmittances of a 50 μm-thick mica substrates. **b,** Micrograph and transmittance of the graphene film on mica substrates shown in Figure 4d.

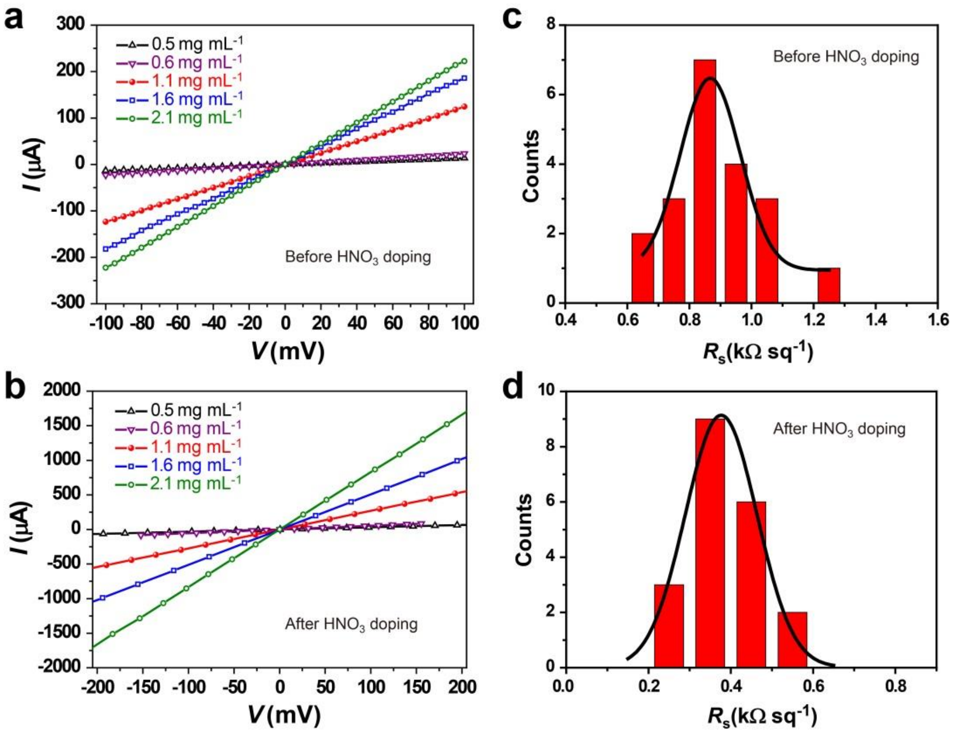

Supplementary Figure 22 | Sheets resistances of the rod-coated graphene films before and after nitric acid doping treatment. a, b, Typical electrical measurements for the graphene films from HBG dispersions with different concentrations. **c, d,** Histograms of sheet resistance distribution of graphene films with ~80% transparency.

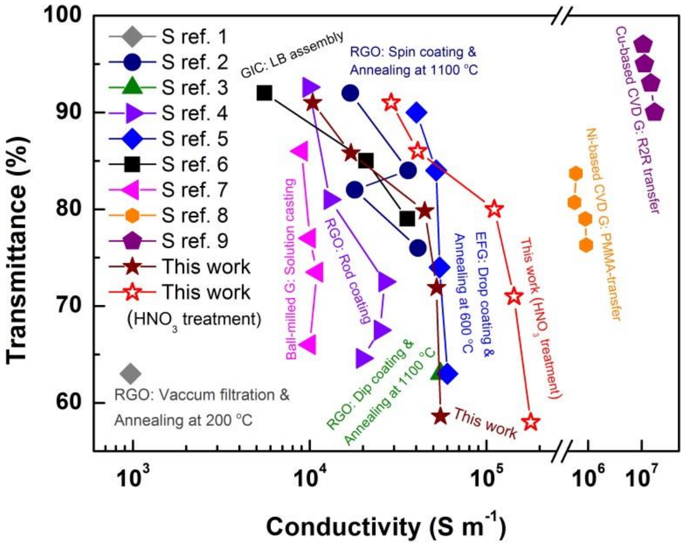

Supplementary Figure 23 | Conductivity versus transmittance for various graphene films prepared by different methods. According to absorbance (2.3%) of monolayer graphene, the film thickness (layer number N) can be calculated from the optical transmittance (T, at 550 nm) of the solution-processed graphene films by the equation '$T = (1- 0.023)^N$'. However, the thickness of HBG films is calculated from the equation ($H_{dry}= H_{wet} \cdot C \cdot \rho^{-1}$) for solution-processed wet films, which has a relative deviation to the theoretical prediction. This is because that the 3D hierarchically porous graphene layers could be not closely packed, leading to an increase of the real film thickness. The as-made graphene films exhibited the highest electrical conductivity (~110,700 S m^{-1}) at the same transmittance (*i.e.* 80%) with regard to those of previously reported solution-based graphene counterparts, it is even close to that of Ni-based CVD graphene films.

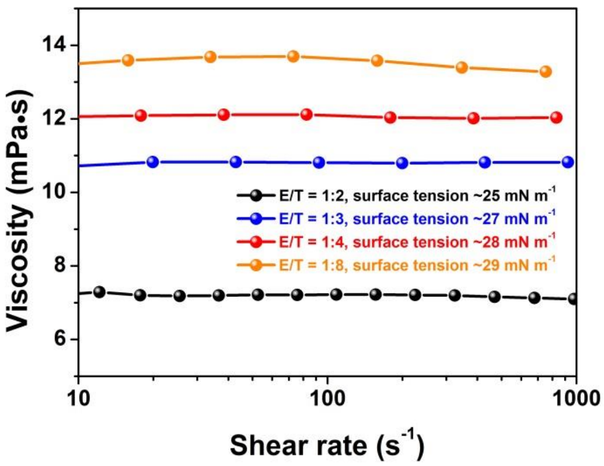

Supplementary Figure 24 | Shear viscosity of the graphene inks (G in ETOH(E)/Terpineol(T)) over the shear rates from 10 to 1000 S^{-1} at 25 °C. The viscosity is measured by a Thermo Scientific Haake RS300 rheometer, and the surface tension is measured by the drop weight method.

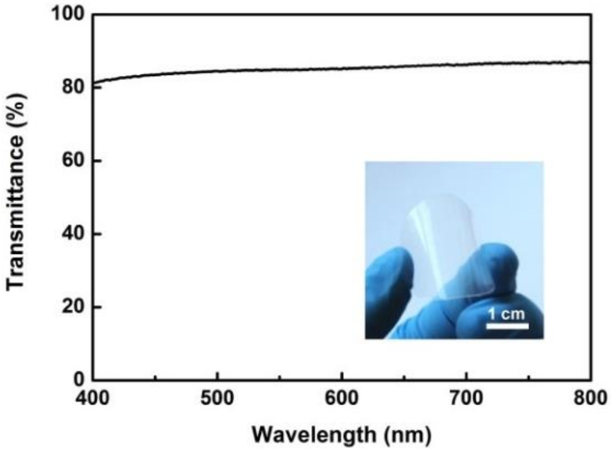

Supplementary Figure 25 | Flexible transparent PET substrates. Transmittance of a 125 μm-thick PET film. Inset: photograph of the corresponding PET films.

Supplementary Table 1 | XPS elemental analyses of HBG and the related materials

Sample	C (%)	O (%)	C/O
GO	67.96	32.04	2.12
RGO	78.58	15.67 (N 5.75)	5.01
Graphite	97.73	2.27	43.05
HBG	96.31	3.69	26.1

Supplementary Table 2 | BET surface areas, pore volumes and average pore diameters of HBG, diatomite and RGO powders

Sample	BET Specific Surface Area ($m^2 g^{-1}$)	Total Pore volume ($cm^3 g^{-1}$)	Average Pore Diameter (nm)
HBG [a]	1137.23	1.94	6.82
Diatomite	9.79	0.01	5.78
RGO	420.95	0.41	3.86

[a] powders grown at methane concentration of 0.6 vol%.

Nitrogen sorption isotherms and textural parameters of the samples were analysed at -196 °C using nitrogen by a Micrometrics ASAP 2010 sorptometer. Prior to analysis, the samples were oven-dried at 150 °C and vacuum-degassed for 12 h at 200 °C. The surface area was calculated using the Brunauer-Emmet-Teller (BET) method based on the adsorption curve in the range of partial pressure (P/P_0) from 0.06 to 0.2, and total pore volume was determined by the amount of single point adsorption at P/P_0 of ca. 0.98. The average pore diameter is calculated by the equation $4V_{\text{total}}/A_{\text{BET}}$.

Supplementary Table 3 | Comparison of HBG and the related RGO and LPEG materials

Sample	Flake size (μm)	Concentration (mg mL)	Conductivity (S m)	Transmittance (%)	Ref.
RGO	0.2-20	0.012 (GO/H_2O)	~350<br>~1000	90%<br>63%	[a]SR1
	<1	1 (GO/H_2O)	200	- (Platelet)	SR10
	~2	0.1 (DMF)	944/7850	- (100 nm films)/-(6.5 μm paper)	SR11
	30-40	2 (H_2O)	1250	-(3 μm films)	SR12
	~0.5	0.5 (H_2O)	7200	- (10 μm films)	SR13
LPEG	~1	0.01 (NMP)	6500	42%	SR14
	0.3-0.8	<0.07 (NMP)	20000	- (1-10μm films)	SR15
HBG	~40	0.5<br>0.6<br>1.1<br>1.6<br>2.1<br>(EtOH/Terpineol)	10400 (28900[b])<br>17100 (40900[b])<br>44800 (110700[b])<br>52200 (143300[b])<br>55000 (178000[b])	91%<br>85.8%<br>79.8%<br>71.9%<br>58.6%	This work
	~40	- (EtOH)	66500	- (1-10μm films)	

[a]SR: Supplementary reference. [b] After HNO_3 doping

Supplementary Table 4 | Comparison of physical properties of HGB and various CVD graphitic porous carbon (CGPC) powders

Sample (Ref.)	Method	Raman spectroscopy (I_D/I_G, I_{2D}/I_G)	Conductivity (S/m)	*Max* A_{BET} increment (times) [a]
CGPC /N-doped CGPC (SR16-19)	Mesoporous silica templated impregnation/carbonization and CVD	1-1.2, no 2D data	397	3.9
GC rods (SR20)	Mesoporous silica-templated chemical vapor/liquid deposition	no data	1360	0.7
CGPC (SR21-23)	X, Y, β-zeolite templated CVD	0.75-0.97, no 2D data	no data	9.2
CGPC (SR24 & 25)	SBA-15 templated CVD	1.4-3.0, no 2D data	20	2.8
Glassy PC (SR26)	Ordered macro-/meso-porous silica templated impregnation/carbonization	0.2-0.8, no 2D data	11-25	1.1
CGPC (SR27)	Mesoporous silica templated impregnation/carbonization	no data	420	1.9
CMK3 (SR28 & 29)	Silica-inverse-opal templated polymerization/ carbonization	0.7-0.8, no 2D data	10	8.9
HBG (This work)	Diatomite templated surface-limited CVD of graphene layers	0.3-0.5, 1.8-0.5 $FWHM_{2D}$: <50 cm^{-1} for ML & BL; >60 cm^{-1} for FL	66500 (Commercial acetylene black: 1125, SR19)	116

[a] Maximal specific-surface-area (A_{BET}) increment of carbons relative to that of silica templates.

Supplementary Table 5 | Comparison of laboratory-scale production rates of graphene powders through various preparation methods

Method (Ref.)	Preparation condition	Estimated production rate in laboratory trials
Chemical oxidation and reduction of graphite (SR30)	Modified Hummer's method: RGO products depending on GO preparation, 100% reduction yield of GO	~0.5 g h^{-1}
Reduction of GO (SR31)	GO reduction by hydroiodic and acetic acid: 2.83g RGO, 40 h (except GO preparation, RGO cleaning and drying processes)	0.07 g h^{-1}
Liquid phase exfoliation of graphite (SR32)	Sonication in NMP: 0.01 g L^{-1}, 10 mL solvent, 30 min	2×10^{-4} g h^{-1}
Liquid phase exfoliation of graphite (SR33)	Shear exfoliation in NMP: 100g L^{-1} graphite, 4500mL NMP, Rotor speed 6000 rpm, >2 min	< 1.44 g h^{-1}
Bottom-up synthesis of few-layer graphene platelets (SR34)	CVD of sodium ethoxide in ethanol	~1 g h^{-1}
This work	CVD on diatomite: ~40 g diatomite, 0.36 g G per batch, 100 min growth (except graphene etching, cleaning and drying processes)	0.22 g h^{-1}

Supplementary references

[1] Eda, G., Fanchini, G. & Chhowalla, M. Large-area ultrathin films of reduced graphene oxide as a transparent and flexible electronic material. *Nature Nanotechnol.* **3**, 270-274 (2008).

[2] Becerril, H. A., *et al.* Evaluation of solution-processed reduced graphene oxide films as transparent conductors. *ACS Nano* **2**, 463-470 (2008).

[3] Wang, X., Zhi, L. & Müllen, K. Transparent, conductive graphene electrodes for dye-sensitized solar cells. *Nano Lett.* **8**, 323-327 (2008).

[4] Wang, J., *et al.* Rod−coating: towards large−area fabrication of uniform reduced graphene oxide films for flexible touch screens. *Adv. Mater.* **24**, 2874-2878 (2012).

[5] Bae, S. Y., *et al.* Large-area graphene films by simple solution casting of edge-selectively functionalized graphite. *ACS Nano* **5**, 4974-4980 (2011).

[6] Park, K. H., *et al.* Exfoliation of non-oxidized graphene flakes for scalable conductive film. *Nano Lett.* **12**, 2871-2876 (2012).

[7] Jeon, I. Y., *et al.* Edge-carboxylated graphene nanosheets via ball milling. *Proc. Natl. Acad. Sci. U. S. A.* **109**, 5588-5593 (2012).

[8] Bae, S., *et al.* Roll-to-roll production of 30-inch graphene films for transparent electrodes. *Nature Nanotechnol.* **5**, 574-578 (2010).

[9] Kim, K. S., *et al.* Large-scale pattern growth of graphene films for stretchable transparent electrodes. *Nature* **457**, 706-710 (2009).

[10] Stankovich, S., *et al*. Synthesis of graphene-based nanosheets via chemical reduction of exfoliated graphite oxide. *Carbon* **45**, 1558-1565 (2007).

[11] Moon, I. K., Lee, J., Ruoff, R. S. & Lee, H. Reduced graphene oxide by chemical graphitization. *Nat. Commun.* **1**, 73 (2010).

[12] Si, Y. & Samulski, E. T. Synthesis of water soluble graphene. *Nano Lett.* **8**, 1679-1682 (2008).

[13] Li, D., Müller, M. B., Gilje, S., Kaner, R. B. & Wallace, G. G. Processable aqueous dispersions of graphene nanosheets. *Nature Nanotechnol.* **3**, 101-105 (2008).

[14] Hernandez, Y., *et al*. High-yield production of graphene by liquid-phase exfoliation of graphite. *Nature Nanotechnol.* **3**, 563-568 (2008).

[15] Paton, K. R., *et al.* Scalable production of large quantities of defect-free few-layer graphene by shear exfoliation in liquids. *Nature Mater.* **13**, 624-630 (2014).

[16] Xia, Y. & Mokaya, R. Synthesis of ordered mesoporous carbon and nitrogen-doped carbon materials with graphitic pore wall via a simple chemical vapor deposition method. *Adv. Mater.* **16**, 1553-1558 (2004).

[17] Xia, Y. & Mokaya, R. Generalized and facile synthesis approach to N-doped highly graphitic mesoporous carbon materials. *Chem. Mater.* **17**, 1553-1560 (2005).

[18] Wang, X., Bozhilov, K. N. & Feng, P. Facile preparation of hierarchically porous carbon monoliths with well-ordered mesostructures. *Chem. Mater.* **18**, 6373-6381 (2006).

[19] Castro-Muñiz, A., Hoshikawa, Y., Kasukabe, T., Komiyama, H. & Kyotani, T. Real understanding

of the nitrogen-doping effect on the electrochemical performance of carbon materials by using carbon-coated mesoporous silica as a model material. *Langmuir* **32**, 2127-2135 (2016).

[20] Yang, Z., Xia, Y., Zhu, Y. & Mokaya, R. Self-assembled ultralarge millimeter-sized graphitic carbon rods grown on mesoporous silica substrate. *Chem. Mater.* **19**, 6317-6322 (2007).

[21] Yang, Z., Xia, Y. & Mokaya, R. Enhanced hydrogen storage capacity of high surface area zeolite-like carbon materials. *J. Am. Chem. Soc.* **129**, 1673-1679 (2007).

[22] Yang, Z., Xia, Y. & Mokaya, R. Aligned N-doped carbon nanotube bundles prepared via CVD using zeolite substrates. *Chem. Mater.* **17**, 4502-4508 (2005).

[23] Yang, Z., Xia, Y., Sun, X. & Mokaya, R. Preparation and hydrogen storage properties of zeolite-templated carbon materials nanocast via chemical vapor deposition: effect of the zeolite template and nitrogen doping. *J. Phys. Chem. B* **110**, 18424-18431 (2006).

[24] Lee, K. T., Ji, X., Rault, M. & Nazar, L. F. Simple synthesis of graphitic ordered mesoporous carbon materials by a solid-state method using metal phthalocyanines. *Angew. Chem. Int. Ed.* **121**, 5771-5775 (2009).

[25] Ji, X., Lee, K. T. & Nazar, L. F. A highly ordered nanostructured carbon-sulphur cathode for lithium-sulphur batteries. *Nature Mater.* **8**, 500-506 (2009).

[26] Wang, Z., Li, F., Ergang, N. S. & Stein, A. Effects of hierarchical architecture on electronic and mechanical properties of nanocast monolithic porous carbons and carbon-carbon nanocomposites. *Chem. Mater.* **18**, 5543-5553 (2006).

[27] Fuertes, A. B. & Alvarez, S. Graphitic mesoporous carbons synthesised through mesostructured silica templates. *Carbon* **42**, 3049-3055 (2004).

[28] Liu, R., *et al.* Triconstituent co-assembly to ordered mesostructured polymer-silica and carbon-silica nanocomposites and large-pore mesoporous carbons with high surface areas. *J. Am. Chem. Soc.* **128**, 11652-11662 (2006).

[29] Schuster, J., *et al.* Spherical ordered mesoporous carbon nanoparticles with high porosity for lithium-sulfur batteries. *Angew. Chem. Int. Ed.* **51**, 3591-3595 (2012).

[30] Liao, K. H., Mittal, A., Bose, S., Leighton, C., Mkhoyan, K. A. & Macosko, C. W. Aqueous only route toward graphene from graphite oxide. *ACS Nano* **5**, 1253-1258 (2011).

[31] Moon, I. K., Lee, J., Ruoff, R. S. & Lee, H. Reduced graphene oxide by chemical graphitization. *Nat. Commun.* **1**, 73 (2010).

[32] Hernandez, Y., *et al.* High-yield production of graphene by liquid-phase exfoliation of graphite. *Nature Nanotechnol.* **3**, 563-568 (2008).

[33] Paton, K. R., *et al.* Scalable production of large quantities of defect-free few-layer graphene by shear exfoliation in liquids. *Nature Mater.* **13**, 624-630 (2014).

[34] Herron, C. R., *et al.* Simple and scalable route for the 'bottom-up' synthesis of few-layer graphene platelets and thin films. *J. Mater. Chem.* **21**, 3378-3383 (2011).
